# Interpersonal violence in the state of Espírito Santo: analysis of mandatory notifications between 2011 and 2018

**DOI:** 10.1590/0034-7167-2023-0081

**Published:** 2024-11-22

**Authors:** Franciéle Marabotti Costa Leite, Luíza Eduarda Portes Ribeiro, Tiffani Matos Oliveira, Evellym Souza Correa, Márcia Regina de Oliveira Pedroso, Dherik Fraga Santos

**Affiliations:** I Universidade Federal do Espírito Santo. Vitória, Espírito Santo, Brazil; II Universidade Federal do Oeste da Bahia. Barreiras, Bahia, Brazil; III Universidade Federal de Catalão. Catalão, Goiás, Brazil

**Keywords:** Exposure to Violence, Violence, Epidemiology, Mandatory Reporting, Health Information Systems, Exposición a la Violencia, Violencia, Epidemiología, Notificación, Sistemas de Información en Salud

## Abstract

**Objective:**

To identify the frequency of notifications of interpersonal violence in Espírito Santo from 2011 to 2018, and the factors associated with this issue.

**Methods:**

This is a cross-sectional study in which all cases of interpersonal violence from the Information System for Notifiable Diseases in the state of Espírito Santo during the period from 2011 to 2018 were analyzed. Absolute and relative frequencies and 95% confidence intervals were calculated, as well as Poisson regression.

**Results:**

During the analyzed period, 27,277 cases were reported in Espírito Santo (P: 75%; 95% CI: 74.5-75.4), being more prevalent among females, children, and the elderly, individuals of black/mixed race, people without disabilities, and residents of urban areas. Regarding the perpetrator, there was a higher prevalence of individuals aged 25 years and older, males, with a current or former intimate partner relationship with the victim, and suspected alcohol use at the time of the aggression. In terms of the characteristics of the aggression, there was a higher prevalence of repeat violence, involving a single aggressor, and occurring in the residence.

**Conclusions:**

The notification of interpersonal violence in Espírito Santo showed a high prevalence and was associated with characteristics of the victim, aggressor, and event. This scenario reinforces the need for interventions such as professional qualification, expansion of intersectoral actions, and reformulation of public policies.

## RESUMEN

**Objetivo:**

Identificar la frecuencia de notificaciones de violencia interpersonal en Espírito Santo, en el período de 2011 a 2018, y los factores asociados a este problema.

**Métodos:**

Se trata de un estudio transversal en el que se analizaron todos los casos de violencia interpersonal del Sistema de Información de Agravios de Notificación en el estado de Espírito Santo durante el período de 2011 a 2018. Se calcularon frecuencias absolutas y relativas y los intervalos de confianza del 95%, así como la regresión de Poisson.

**Resultados:**

En el período analizado, se notificaron 27.277 casos en Espírito Santo (P: 75%; IC95%: 74,5-75,4), siendo más prevalentes entre individuos del sexo femenino, niños y ancianos, individuos de raza/color negro/mestizo, personas sin discapacidad y residentes de la zona urbana. En cuanto al perpetrador, se verificó una mayor prevalencia de personas de 25 años o más, hombres, con vínculo con la víctima como pareja íntima actual o ex, y con sospecha de uso de alcohol en el momento de la agresión. En relación con las características de la agresión, se observó una mayor prevalencia de violencias repetitivas, con la participación de un solo agresor y la residencia como lugar de ocurrencia.

**Conclusiones:**

La notificación de violencia interpersonal en Espírito Santo presentó alta prevalencia y estuvo asociada a características de la víctima, agresor y evento. Este escenario refuerza la necesidad de intervenciones como la cualificación profesional, la ampliación de acciones intersectoriales y la reformulación de políticas públicas.

## INTRODUCTION

Violence is a social, historical, multicausal, and complex phenomenon that accompanies all human experience ^(1)^. According to the World Health Organization (WHO), it can be described as the use of physical force or power, real or threatened, against oneself, another individual, or a group or community, that results in or has a high likelihood of resulting in injury, psychological harm, death, developmental disability, or deprivation ^(1,2)^.

One type of violence is interpersonal violence, considered a priority public health issue worldwide and widely discussed by various sectors of society due to its impact on public safety indicators, its influence on individuals’ daily lives, and the constant presence of victims in health services ^(3)^. This type of violence can be defined as occurring between family members, intimate partners, friends, acquaintances, and strangers, including child maltreatment, youth violence (including gang-related violence), violence against women, and elder abuse ^(3)^.

Interpersonal violence is subdivided into intrafamilial/intimate partner violence, generally occurring in the domestic environment, although not limited to this setting, and community violence, where individuals are not related, may or may not know each other, and usually occurs outside the home. It is the third leading cause of death among adolescents globally, although its prominence varies by region ^(4)^.

This type of violence causes nearly one-third of all deaths among male adolescents in lowand middle-income countries in the Americas ^(5)^. Additionally, it is estimated that in 2000, 520,000 people worldwide died as a result of interpersonal violence, representing a rate of 8.8 people per 100,000 ^(4)^.

In Brazil, regarding intrafamilial violence, there has been a significant increase since the late 1980s, occupying the first place among the causes of death in the young population (15 to 24 years old), particularly among young black individuals living in the peripheries and metropolitan areas of urban centers. It affects at least one in three adolescents aged 15 to 19 years, whether due to emotional, physical, psychological, and/or sexual violence perpetrated by a husband or partner. In 2016, 62,517 intentional violent deaths and 49,497 rapes were recorded in the country ^(5,6)^.

In addition to prevalence, another consideration in the study of violence is its complexity. The emergence of violent acts involves individual, relational, cultural, and environmental factors, creating a direct influence between the individual and their environment, which underscores the importance of local studies ^(2)^.

Thus, annually, millions of people lose their lives, and many others sustain non-fatal injuries due to domestic and community violence. Victims suffer from various adverse effects, including anxiety, depression, substance abuse, post-traumatic stress disorder (PTSD), and suicide attempts ^(3)^, in addition to developing physical health conditions (cardiovascular disease, chronic pain, sleep disorders, gastrointestinal problems, sexually transmitted infections, traumatic brain injury) ^(7)^.

It is also known that this number of cases is lower than what actually occurs due to underreporting. This is because of the fear and shame of reporting by the victims, as the aggressor is often someone in their close circle, and also due to the culture of normalization of violence present in society ^(8)^.

Therefore, it is imperative that primary care teams, who are geographically closer to families and involved in individual and collective health actions, be trained. These teams are in a better position to identify situations of violence through welcoming, care (diagnosis, treatment, and support), reporting cases, and referral to care and social protection networks ^(9)^.

It is essential to contextualize that in Espírito Santo, according to data from an Epidemiological Bulletin, there was an observed increase of 1017% in the number of annual violence notifications from 2011 to 2018, rising from 855 cases reported in 2011 to 9549 forms filled out in 2018 alone, demonstrating an average annual growth in notifications of 145%. The total number of municipalities that reported violence increased from 28% in 2011 to 94% in 2018. However, four municipalities (Pancas, Bom Jesus do Norte, Águia Branca, and Ibitirama) did not report any cases during this period, despite having several trained professionals, especially at the end of 2018 ^(10)^.

Despite the increase in reported cases over the years, there was no proportional increase in the availability of referral services for attending to individuals in situations of violence in the health sector in Espírito Santo. Only two municipalities, Vila Velha and Vitória, have their own reference services that provide specialized care to the population experiencing sexual violence and violations of children’s and adolescents’ rights. The Program for Assistance to Victims of Sexual Violence (PAVIVIS) is the only state reference service, located at the University Hospital in Vitória-ES, which restricts its target audience to those over 12 years of age due to limitations in the number of professionals, logistics, and physical space. This service is a university extension project of the Federal University of Espírito Santo (UFES) and is one of the two accredited for legal abortion procedures, being the only one in the metropolitan and southern regions. The other service is located in the municipality of Colatina, in the central-west region of ES. There are still no health services equipped to collect evidence of sexual violence, complicating the structuring of the chain of custody^(10)^.

Given the above, in light of the health damages caused by interpersonal violence, the need for studies on this violence is strengthened. Such studies could assist in the professional qualification process and the restructuring of public policies in the face of the scenario of addressing interpersonal violence. Therefore, the present study aimed to identify the frequency of notifications of interpersonal violence in Espírito Santo from 2011 to 2018 and the factors associated with this issue.

## OBJECTIVE

To identify the frequency of notifications of interpersonal violence in Espírito Santo from 2011 to 2018 and the factors associated with this issue.

## METHODS

### Ethical Aspects

The data analyzed in this study come from the Interpersonal and Self-Inflicted Violence Notification/Investigation Forms, filled out by professionals from private and public health services and digitized in the Information System for Notifiable Diseases (SINAN in Portuguese). Patient consent was waived because the database was provided by the Epidemiological Surveillance sector of the Espírito Santo State Health Department. This study uses secondary data; however, because it contains nominal identification information in the database provided by the mentioned Epidemiological Surveillance, the study was approved by the Research Ethics Committee of the Federal University of Espírito Santo in 2018, following National Health Council Resolution No. 466/12.

### Study Design and Setting

A cross-sectional study was conducted. The EQUATOR Network guidelines were applied using the Strengthening the Reporting of Observational Studies in Epidemiology (STROBE) tool ^(11)^. The study setting was the state of Espírito Santo, which has a population of 3,514,952, a Human Development Index (HDI) of 0.740, and a population density of 76.25 inhabitants/km^2 (12)^.

### Population, Period, Inclusion and Exclusion Criteria

This study included all reported cases of interpersonal violence in the state of Espírito Santo from 2011 to 2018, based on data recorded in the SINAN provided by the Espírito Santo Epidemiological Surveillance. The study period was chosen because violence was included as a notifiable condition starting in 2011 ^(13)^. No cases were excluded; all compulsory notification forms of interpersonal violence during the described period in Espírito Santo were analyzed. The data recorded on the SINAN notification form and provided by the Espírito Santo State Health Department originate from services provided by public and private health services. The understanding of the characteristics analyzed in the compulsory notification form follows the guidelines of the Interpersonal and Self-Inflicted Notification Manual ^(13)^.

### Variables

The dependent variable in this study was interpersonal violence (analyzed dichotomously - yes/no), regardless of its nature. In this study, interpersonal violence is defined as specified in the Interpersonal and Self-Inflicted Notification Manual, which distinguishes between two types: domestic/intrafamilial violence, identified as occurring between intimate partners or family members either within or outside the home, and extrafamilial/community violence, which occurs in the social environment involving both acquaintances and strangers ^(13)^. The concept encompasses various forms of interpersonal violence such as physical, psychological/moral, sexual, torture, human trafficking, child labor, legal intervention, financial/economic, and neglect/abandonment ^(13)^.

As independent variables, the characteristics of the victim, the aggressor, and the aggression were analyzed. The victim’s characteristics included sex (male; female), age group (in years: 0 to 9; 10 to 19; 20 to 59; 60 or more), race/color (white; black/mixed), presence of disabilities/disorders (no; yes), and area of residence (urban/peri-urban; rural).

Regarding the characteristics of the aggressor, the variables evaluated were age group (in years: 0 to 24; 25 or more), sex (male; female), relationship to the victim (current or former intimate partner; family member; acquaintance; stranger), and suspicion of alcohol use (no; yes).

For the characteristics of the aggression, the variables analyzed were the number of individuals involved (one; two or more), the location of occurrence (residence; public space; others), the presence of a history of repeated violence (no; yes), and whether the victim was referred to other services (no; yes).

### Analysis of Results and Statistics

Before the analyses, the data underwent a qualification process following the guidelines of the Interpersonal and Self-Inflicted Notification Manual ^(13)^. Cases with blank or ignored data were excluded from the analyses, resulting in a varying total number of individuals depending on the characteristic studied. Subsequently, the data were processed using the statistical package Stata, version 14.0, and the results were presented as absolute and relative frequencies, as well as 95% confidence intervals. For the bivariate analysis, Pearson’s Chi-Square Test was used, and for the multivariate analysis, Poisson Regression with prevalence ratio estimates was applied. A hierarchical model was used, considering the victim’s characteristics as the first level and the variables related to the aggressor and the event as the second level. Variables entered the model if they met the criterion of p < 0.20 in the bivariate analysis, and were retained if p < 0.05. The variables “relationship with the victim” and “number of individuals involved” were not included in the bivariate and multivariate analyses because the comparison group consists of notifications of self-inflicted violence.

## RESULTS

From 2011 to 2018, in Espírito Santo, 27,277 cases of interpersonal violence were reported, representing a frequency of 75% (95% CI: 74.5-75.4) among all notifications made during the period (data not presented in the table).

Regarding the characterization of the victims, 75.0% were women (n=20,449), adults (59.4%; n=16,191), of black/mixed race (71.0%; n=16,928), those without disabilities or disorders (90.7%; n=21,362), and those residing in urban/peri-urban areas (90.3%; n=24,129). Concerning the aggressor, the majority were aged 25 years or older (68.9%; n=10,091), men (82.9%; n=19,054), with a current or former intimate partner relationship with the victim (40.6%; n=9,811), and without suspicion of alcohol use during the event (54.5%; n=7,647).

As for the aggression, it was observed that the majority were committed by a single aggressor (80.5%; n=19,628), occurring in residences (66.6%; n=15,853), being repetitive (54.5%; n=11,293), and, in most cases (84.9%; n=21,895), the victims were referred to other services (Table 1).

**Table 1:** Characteristics of reported cases of interpersonal violence. Espírito Santo, Brazil, 2011-2018

Variables	n	%	IC 95%^*^
Sex			
Male	6828	25.0	24.5-25.6
Female	20449	75.0	74.5-75.5
Age Group			
0 to 9 years	3060	11.2	10.9-11.6
10 to 19 years	6348	23.3	22.8-23.8
20 to 59 years	16191	59.4	58.8-59.9
60 years and older	1678	6.1	5.9-6.4
Race/Color			
White	6911	29.0	28.4-29.6
Black/Mixed	16928	71.0	70.4-71.6
Disabilities/Disorders			
No	21362	90.7	90.3-91.1
Yes	2191	9.3	8.9-9.7
Area of Residence			
Urban/Peri-urban	24129	90.3	89.9-90.6
Rural	2603	9.7	9.4-10.1
Age Group of Aggressor			
0 to 24 years	4546	31.1	30.3-31.8
25 years and older	10091	68.9	68.2-69.7
Sex of Aggressor			
Male	19054	82.9	82.4-83.4
Female	3938	17.1	16.7-17.6
Relationship with the Victim			
Current or former intimate partner	9811	40.6	39.9-41.2
Family member	5132	21.2	20.7-21.7
Acquaintance	5772	23.9	23.3-24.4
Stranger	3475	14.3	13.9-14.8
Suspicion of Alcohol Use			
No	9163	54.5	53.8-55.3
Yes	7647	45.5	44.7-46.3
Number of Aggressors			
One	19628	80.5	80.0-81.0
Two or more	4762	19.5	19.0-20.0
Location of Occurrence			
Residence	15853	66.6	66.0-67.2
Public space	5121	21.5	21.0-22.1
Others	2823	11.9	11.5-12.3
Repeat Violence			
No	9445	45.5	44.9-46.2
Yes	11293	54.5	53.8-55.1
Referral			
No	3900	15.1	14.7-15.6
Yes	21895	84.9	84.4-85.3

* *IC95%: 95% Confidence Interval; Source: Information System for Notifiable Diseases (SINAN), from 2011 to 2018.*

In the bivariate analysis, the outcome was associated with most of the independent variables studied, except for the sex of the victim, area of residence, and repetition of the event (Table 2).

**Table 2:** Bivariate analysis of the distribution of characteristics according to notifications of interpersonal violence. Espírito Santo, Brazil, 2011-2018

Variables	n	%	IC 95%^*^	*p* value
Sex				
Male	6828	75.2	74.3-76.1	0.493
Female	20449	74.9	74.4-75.4	
Age group				
0 to 9 years	3060	98.8	98.4-99.1	<0.001
10 to 19 years	6348	72.1	71.1-73.0	
20 to 59 years	16191	71.9	71.3-72.4	
60 years and older	1678	85.8	84.2-87.3	
Race/color				
White	6911	71.3	70.4-72.2	<0.001
Black/mixed	16928	78.0	77.4-78.6	
Disabilities/disorders				
No	21362	82.6	82.1-83.0	<0.001
Yes	2191	48.2	46.7-49.6	
Area of residence				
Urban/peri-urban	24129	74.8	74.3-75.3	0.217
Rural	2603	75.8	74.3-77.2	
Age group of the aggressor				
0 to 24 years	4546	55.0	53.9-56.1	<0.001
25 years and older	10091	70.5	69.8-71.3	
Sex of the aggressor				
Male	19054	88.9	88.5-89.3	<0.001
Female	3938	38.0	37.1-39.0	
Suspicion of alcohol use				
No	9163	65.5	64.7-66.3	<0.001
Yes	7647	86.2	85.5-86.9	
Location of occurrence				
Residence	15853	68.7	68.1-69.3	<0.001
Public space	5121	92.5	91.8-93.2	
Others	2823	85.3	84.1-86.5	
Repeat violence				
No	9445	74.7	74.0-75.5	0.160
Yes	11293	75.5	74.8-76.1	
Referral				
No	3900	69.6	68.4-70.8	<0.001
Yes	21895	76.1	75.6-76.6	

* *IC95%: 95% Confidence Interval; Source: Information System for Notifiable Diseases (SINAN), from 2011 to 2018.*

In the multivariate analysis, after controlling for confounding factors, children and the elderly had 29% and 25% higher prevalence of interpersonal violence notifications, respectively, compared to adolescents. Another finding was a higher frequency of notifications among individuals of black/mixed race (PR: 1.07; 95% CI: 1.06-1.09), compared to those who identified as white. Additionally, it was noted that notifications of interpersonal violence were 69% higher in people without disabilities. Regarding the perpetrator, there was a higher prevalence among individuals aged 25 years or older (PR: 1.15; 95% CI: 1.13-1.18), men were twice as likely (PR: 2.09; 95% CI: 2.01-2.17), and those suspected of alcohol use at the time of the aggression (PR: 1.12; 95% CI: 1.10-1.14). In terms of the location of the violence, public spaces were the most common locations for reported incidents (PR: 1.32; 95% CI: 1.29-1.35), and repeat violence was also a significant factor (PR: 1.09; 95% CI: 1.07-1.11) (Table 3).

**Table 3:** Multivariate model with crude and adjusted prevalence ratios of variables associated with notifications of interpersonal violence. Espírito Santo, Brazil, 2011-2018

Variables	Crude analysis			Adjusted analysis		
	PR^*^	95%CI^†^	*p* value	PR^*^	95%CI^†^	*p* value
Age group						
0 to 9 years	1.37	1.35-1.39	<0.001	1.29	1.27-1.30	<0.001
10 to 19 years	1.0			1.0		
20 to 59 years	0.99	0.98-1.01		1.04	1.02-1.05	
60 years and older	1.19	1.17-1.22		1.25	1.22-1.28	
Race/color						
White	1.0		<0.001	1.0		<0.001
Black/mixed	1.10	1.08-1.11		1.07	1.06-1.09	
Disabilities/disorders						
No	1.72	1.66-1.77	<0.001	1.69	1.64-1.75	<0.001
Yes	1.0			1.0		
Age group of the aggressor						
0 to 24 years	1.0		<0.001	1.0		<0.001
25 years and older	1.28	1.25-1.31		1.15	1.13-1.18	
Sex of the aggressor						
Male	2.34	2.28-2.40	<0.001	2.09	2.01-2.17	<0.001
Female	1.0			1.0		
Suspicion of alcohol use						
No	1.0		<0.001	1.0		<0.001
Yes	1.32	1.30-1.34		1.12	1.10-1.14	
Location of occurrence						
Residence	1.0		<0.001	1.0		<0.001
Public space	1.35	1.33-1.36		1.32	1.29-1.35	
Others	1.24	1.22-1.26		1.22	1.19-1.26	
Repeat violence						
No	1.0		0.160	1.0		<0.001
Yes	1.01	0.99-1.02		1.09	1.07-1.11	

*RP: Prevalence Ratio; †95% CI: 95% Confidence Interval; Source: Information System for Notifiable Diseases (SINAN), from 2011 to 2018.*

## DISCUSSION

The present study identified a high frequency of notifications of interpersonal violence in Espírito Santo from 2011 to 2018, corroborating research conducted in Paraná, where the prevalence of this issue also represented the majority of notifications (90%) compared to self-inflicted violence (10%) ^(14)^. In absolute numbers, the study conducted in Paraná found that the population most affected by interpersonal violence was children. In the present study, the highest occurrence of interpersonal violence notifications was among adult women. However, from a statistical association perspective, children aged 0-9 years had a 29% higher prevalence compared to adolescents aged 10-19 years, while adults aged 20-59 years had a 4% higher prevalence compared to adolescents ^(14)^.

There is also a higher prevalence of notifications of interpersonal violence against children. It is important to consider that this population is more dependent on their families, which can either provide support and safety or neglect their responsibilities, depriving the child of their rights and thus impairing their physical, social, behavioral, emotional, and cognitive development ^(15)^. In the study conducted in Paraná, it was observed that the most frequent type of interpersonal violence reported among children was neglect/abandonment ^(14)^. A study analyzing notifications of violence in Espírito Santo found that parents/step-parents were the most prevalent aggressors in cases of neglect/abandonment ^(16)^, and the main reasons for violence against individuals in this age group are low family income, low educational level, poverty, and lack of family planning ^(17)^.

The present research found a higher prevalence of notifications of interpersonal violence among individuals of black/mixed race, which is consistent with a Brazilian study ^(18)^. The black population is the primary victim of socioeconomic inequities, which are attributed to social inequalities, prejudice, and discrimination present in society. This context becomes a fertile ground for the occurrence of violence ^(19)^. An important perspective on the relationship between violence and the black population is the theoretical lens of intersectionality, a way of observing the structural and dynamic consequences of the relationship between two or more axes of subordination, such as the interaction of racism with other factors. This intersectional view makes the social vulnerabilities that expose the black population to a higher frequency of violent situations more evident ^(20)^.

It is interesting to note that individuals without disabilities were the most prevalent among the reported cases in this study. Data from the 2017 VIVA survey highlighted a frequency of 95.7% of assaults perpetrated against people without disabilities ^(21)^. However, it is worth mentioning the challenges involved in identifying and recognizing violent acts committed against people with disabilities. Between 2009 and 2013, Brazil saw a 7.1% increase in reported cases of interpersonal violence against this population ^(22)^. Difficulties related to the definition of the term “disability,” as well as the lack of visibility of the victim due to their limitations and the power dynamics established by family members and caregivers, contribute to the distancing from protective measures and reporting ^(23)^.

This study identified a higher occurrence of violence involving adult male aggressors. This finding was also observed in a study that analyzed notifications of interpersonal violence across Brazil from 2015 to 2019, which found a prevalence of 54% male aggressors and 45% adult aggressors ^(17)^. In this context, it is important to consider that men who commit violence often uphold a discourse of dominance over women, the provision of the household, family leadership, and exaggerated sexuality. These social attributes of masculinity fuel the practice of domestic violence. The men who commit violence cited these characteristics as intrinsic and indispensable to what it means to be a man, suggesting that any act of insubordination by a woman or girl that threatens this model of masculinity is considered a trigger for violence ^(24)^.

Alcohol use by the aggressor at the time of the aggression was associated with a 12% increase in the prevalence of interpersonal violence compared to those who did not consume alcohol, a finding also seen in another study ^(25)^. According to the Alcohol and Drugs Research Unit at the Federal University of São Paulo, alcohol consumption by the aggressor, even in non-abusive forms, plays a significant role in the perpetuation of violence ^(26)^. Furthermore, alcohol plays an important role in physiological disinhibition and also affects the expectation of others to accept such behaviors, often resulting in violent behavior ^(27,28)^. Alcohol use is also associated with greater severity of intimate partner violence consequences, a finding that is consistent with both national and international studies ^(27-29)^.

Regarding the location of the reported cases, public spaces had the highest overall frequency of reported interpersonal violence cases, corroborating the findings of a study that analyzed 4,406 cases of violence that occurred in Brazilian capitals ^(30)^. However, this same study points out that there is differentiation in the profile of the location of occurrence when a stratified analysis is performed, especially concerning gender and age.

Public spaces (streets, bars, and other locations) represent the main setting for violent events, especially involving male aggressors and victims. The home is where most violence against women, children, adolescents, and the elderly occurs, as it is where they spend most of their time and where most aggressors live. Any family member can become a victim or aggressor under certain circumstances ^(30)^.

After a simple correspondence analysis, public spaces remained associated with violent events rather than residences ^(31)^. Nevertheless, it is important to highlight the greater vulnerability of victims in domestic environments. These victims may remain silenced by their aggressors and only come to attention when exposed in external environments, since violence is often reported when the service has contact with the victim. This is a limitation, as not all cases have contact with a notifying service (public or private health services, schools) ^(2,23)^.

It is worth noting the repetitive nature of the reported cases of interpersonal violence found in this study, similar to findings in studies by Leite ^(32)^. Repetition is often associated with close contact with the aggressor, often a family member in the residence, which can contribute to delays in seeking health services ^(33)^. Additionally, when it comes to psychological violence and neglect, it becomes difficult to record due to its subjectivity, contributing to the recurrence ^(34)^. Furthermore, the victim must deal with the embarrassment, fear of humiliation, and misunderstanding, often leading to self-blame, which prevents reporting and increases the chances of violence recurrence. Consequently, by continuing in this violent context, victims are at greater risk of developing eating disorders, alcoholism, drug abuse, post-traumatic stress disorder, depression, anxiety, phobias, panic, and low self-esteem ^(33,35)^.

As a complex problem that requires the involvement of various sectors for its resolution, healthcare needs to act in an interconnected network, making multidisciplinary teamwork essential. Regarding nursing, it has a crucial tool for detecting cases of violence: consultations, during which anamnesis and physical examinations are conducted. It is important to clarify that during anamnesis, certain aspects of speech and behavior can be indicators of suspected violence for health professionals ^(36)^.

Thus, the primary responsibilities of nurses, especially in primary care, are as follows: planning health promotion actions, preventing violent actions and health issues, and finally, interacting with other teams to ensure the victim receives comprehensive care ^(37)^. Additionally, it is the responsibility of health professionals to identify and report suspected or confirmed cases of violence. Continuous training on this topic and the provision of tools that facilitate and enhance the reporting process, such as an online system, can be crucial for improving reporting as an important aspect of the care continuum ^(5)^.

In this educational and awareness process, nurses, as members of the health team and knowledge multipliers, are of utmost importance. By promoting health education within the population, they will provide the necessary knowledge to foster interest and demystify the fear related to reporting ^(38)^.

Furthermore, it is essential to highlight the effectiveness of a multidisciplinary approach to cases of individuals experiencing interpersonal violence. In this approach, an important factor is that professionals are trained to use the information acquired and observed during the identification and post-identification of violence to avoid committing further violence against the victim’s vulnerability. This applies to both the health needs of individuals experiencing violence and the intersectoral care that aims to provide the necessary protection and care, varying according to the complexities and particularities of each case ^(39)^. The lack of preparation of the multidisciplinary team in identifying and managing cases of violence can contribute to the perpetuation of the violent situation, favoring the maintenance or worsening of the scenario, thus exposing the individual to a context that promotes the repetition of violent acts ^(40)^.

Given the relevance of violence reporting, it is worth emphasizing that reporting is one of the key elements of the Care Line for comprehensive health care for individuals, whether children, adults, elderly, men, women, or other family members, in situations of violence. This line of care, in addition to monitoring activities and preventing violence, also acts in reception, care, prophylactic measures, treatment, follow-up in the care network, social protection, and promotion of health and a culture of peace ^(41-42)^. These processes adhere to legislation that provides assistance and rights for specific groups, such as the Child and Adolescent Statute, the Elderly Statute, the Statute of Persons with Disabilities, technical norms from the Ministry of Health, among others ^(41)^. Thus, the importance of reporting as a tool for care and rights protection is reiterated.

### Contributions to the Field

The findings of this study may assist in the development or restructuring of public policies to address different forms of interpersonal violence. Likewise, the results can broaden the health professionals’ perspective on caring for the population most vulnerable to interpersonal violence, encouraging prevention and promotion interventions against the occurrence of this phenomenon, as well as reinforcing the importance of professional training, thereby improving data entry, system launch, and reducing the incompleteness of the notification forms.

## CONCLUSIONS

In Espírito Santo, from 2011 to 2018, the results show a high number of reported cases of interpersonal violence. However, the presence of underreporting must be considered, which would result in a higher number of victims, a worrying fact that highlights the importance of qualified professionals capable of screening for signs and symptoms suggestive of violence during health service consultations, and consequently, reporting and managing the cases.

Another point to highlight is the greater vulnerability of children and the elderly to interpersonal violence, as well as the fact that men are the main perpetrators. These findings reveal how violence is tied to power inequality relationships, making it highly important to address the topic of gender equality in various individual development scenarios, such as family, school, and health services.

Finally, and equally important, is the observation that victimization was recurrent. This finding is extremely concerning as it indicates how early detection of violence is still flawed and how victims remain in this cycle repeatedly. Health services and other assistance spaces must strive for early detection of violence and, thus, facilitate the integration of the victim into the care and protection network.

Therefore, based on the data found in this study, it is evident that there is a need to strengthen intersectoral action networks for the protection and care of victims, considering the multidimensional nature of violence, in addition to strengthening public policies to ensure their effectiveness and the protection of the most vulnerable segments of society.
