# Supplementary material for: Interpersonal violence in the state of Espírito Santo: analysis of mandatory notifications between 2011 and 2018
Source: Rev Bras Enferm. 2024 Nov 22;77(5):e20230081. doi: 10.1590/0034-7167-2023-0081 (PMC11653869; doi:10.1590/0034-7167-2023-0081)
Supplement: Translation

# Translation: Violência interpessoal no estado do Espírito Santo: análise das notificações compulsórias entre 2011 e 2018

**DOI:** 10.1590/0034-7167-2023-0081pt

**Authors:** Franciéle Marabotti Costa Leite, Luíza Eduarda Portes Ribeiro, Tiffani Matos Oliveira, Evellym Souza Correa, Márcia Regina de Oliveira Pedroso, Dherik Fraga Santos

**Affiliations:** I Universidade Federal do Espírito Santo. Vitória, Espírito Santo, Brasil; II Universidade Federal do Oeste da Bahia. Barreiras, Bahia, Brasil; III Universidade Federal de Catalão. Catalão, Goiás, Brasil

**Keywords:** Exposição à, Violência, Violência, Epidemiologia, Notificação, Sistemas de Informação em Saúde

## Abstract

**Objetivo:**

Identificar a frequência de notificações de violência interpessoal no Espírito Santo, no período de 2011 a 2018, e os fatores associados a esse agravo.

**Métodos:**

Trata-se de um estudo transversal, no qual foram analisados todos os casos de violência interpessoal do Sistema de Informação de Agravos de Notificação no estado do Espírito Santo no período de 2011 a 2018. Foram calculadas frequências absolutas e relativas e os intervalos de confiança de 95%, bem como a regressão de Poisson.

**Resultados:**

No período analisado, foram notificados 27.277 casos no Espírito Santo (P: 75%; IC95%: 74,5-75,4), sendo mais prevalente entre indivíduos do sexo feminino, crianças e idosos, indivíduos de raça/cor preta/parda, pessoas sem deficiência e residentes da zona urbana. Quanto ao perpetrador, verificou-se uma maior prevalência de pessoas com 25 anos ou mais, homens, apresentando vínculo com a vítima de parceiro íntimo atual ou ex, e com suspeita de uso de álcool no momento da agressão. Em relação às características da agressão, ocorreu uma maior prevalência de violências de repetição, com envolvimento de um agressor e a residência como local de ocorrência.

**Conclusões:**

A notificação de violência interpessoal no Espírito Santo apresentou alta prevalência e esteve associada a características da vítima, agressor e evento. Esse cenário fortalece a necessidade de intervenções, como as de qualificação profissional, de ampliação de ações intersetoriais e de reformulações de políticas públicas.

## INTRODUÇÃO

A violência é um fenômeno social, histórico, multicausal e complexo, que acompanha toda a experiência humana ^(1)^. Segundo a Organização Mundial de Saúde (OMS), pode ser descrita como a utilização da força física ou do poder, real ou ameaçado, contra si próprio, contra outro indivíduo, ou contra um grupo ou uma comunidade, que acarrete ou tenha qualquer possibilidade de ocasionar lesão, dano psicológico, morte, deficiência de desenvolvimento ou privação ^(1,2)^.

Uma das tipologias da violência é a violência interpessoal, considerada um problema prioritário de saúde pública em todo o mundo e amplamente discutida por vários setores da sociedade, em razão do reflexo nos indicadores de segurança pública, pela influência no cotidiano dos indivíduos, assim como pela presença constante de vítimas nos serviços de saúde ^(3)^. Essa violência pode ser definida como a que ocorre entre membros da família, parceiros íntimos, amigos, conhecidos e desconhecidos, incluindo maus-tratos à criança, violência juvenil (incluindo aquelas associadas a gangues), violência contra mulheres e violência contra o idoso ^(3)^.

A violência interpessoal é subdividida em: intrafamiliar/parceiros íntimos, geralmente ocorrendo em ambiente doméstico, apesar de não se limitar a esse lugar, e comunitária, em que os indivíduos não estão relacionados entre si, podendo se conhecer ou não, ocorrendo normalmente fora da residência; sendo a terceira principal causa de morte entre adolescentes globalmente, embora sua proeminência varie de acordo com a região do mundo ^(4)^.

Essa violência causa quase um terço de todas as mortes de adolescentes do sexo masculino em países de baixa ou média renda da região das Américas ^(5)^. Além disso, estima-se que, no ano de 2000, 520.000 pessoas morreram no mundo em decorrência da violência interpessoal, o que representa uma taxa de 8,8 pessoas a cada 100.000 ^(4)^.

No Brasil, no que se refere à violência intrafamiliar, há um crescimento expressivo desde o fim da década de 1980, ocupando desde então o primeiro lugar entre as causas de morte na população jovem (15 a 24 anos), principalmente de jovens negros, residentes nas periferias e áreas metropolitanas dos centros urbanos. Afeta pelo menos um a cada três adolescentes entre 15 a 19 anos, quer seja por violência emocional, física, psicológica e/ou sexual perpetrada pelo marido ou parceiro. Em 2016, foram registradas 62.517 mortes violentas intencionais e 49.497 estupros no país ^(5,6)^.

Além das prevalências, outro fator de ponderação no estudo da violência é a sua complexidade. O surgimento do ato violento engloba fatores individuais, relacionais, culturais e ambientais, fazendo com que haja uma influência direta entre o indivíduo e o meio em que ele vive, trazendo ainda mais importância para estudos em âmbito local ^(2)^.

Assim, anualmente, milhões de pessoas perdem a vida, e muitas outras sustentam ferimentos não fatais decorrentes de violência doméstica e comunitária. As vítimas sofrem vários efeitos adversos, incluindo ansiedade, depressão, abuso de substâncias psicoativas, transtorno de estresse pós-traumático (TEPT) e tentativa de suicídio ^(3)^, além de desenvolver condições de saúde física (doença cardiovascular, dor crônica, distúrbios do sono, problemas gastrointestinais, infecções sexualmente transmissíveis, lesão cerebral traumática) ^(7)^.

Sabe-se também que esse quantitativo de casos está abaixo do que realmente acontece devido à subnotificação dos casos. Isso se deve ao medo e vergonha da denúncia por parte das vítimas, já que muitas vezes o agressor é uma pessoa do seu convívio íntimo, e também pela cultura de naturalização da violência presente na sociedade ^(8)^.

Dessa forma, é imperativo que, principalmente, as equipes da atenção primária, por se encontrarem geograficamente mais próximas das famílias e pelo envolvimento dos profissionais com as ações de saúde individual e coletiva, sejam capacitadas, uma vez que esses têm uma maior possibilidade de identificar as situações de violência, por meio do acolhimento, atendimento (diagnóstico, tratamento e cuidados), notificação dos casos e encaminhamento para a rede de cuidados e de proteção social ^(9)^.

É essencial contextualizar que, no Espírito Santo, segundo dados de um Boletim Epidemiológico, foi observado que de 2011 a 2018 ocorreu um aumento de 1017% no número de notificações de violência por ano, passando de 855 casos notificados em 2011 para 9549 fichas preenchidas somente em 2018, demonstrando uma média de crescimento das notificações de 145% ao ano. A quantidade total de municípios que realizaram notificações de violência saltou de 28% em 2011 para 94% em 2018. No entanto, quatro municípios (Pancas, Bom Jesus do Norte, Águia Branca e Ibitirama) não apresentaram notificações nesse período, apesar de vários profissionais capacitados, sobretudo no final de 2018 ^(10)^.

Apesar desse aumento dos casos notificados ao longo dos anos, não ocorreu um aumento proporcional da oferta dos serviços de referência para atendimento às pessoas em situação de violência no setor saúde no Espírito Santo. Somente dois municípios, Vila Velha e Vitória, possuem serviços próprios de referência que prestam atendimento especializado à população em situação de violência sexual e violação de direitos infantojuvenis. Já o Programa de Atendimento à Vítima de Violência Sexual (PAVIVIS) é o único serviço de referência estadual, localizado no Hospital Universitário em Vitória-ES, que restringe o público-alvo à faixa etária acima de 12 anos, devido a limitações de quantitativo de profissionais, logística e espaço físico. Este serviço é um projeto de extensão universitária da Universidade Federal do Espírito Santo (UFES) e é um dos dois credenciados para a interrupção da gravidez prevista em lei, sendo o único nas regiões metropolitana e sul. O outro serviço está localizado no município de Colatina, na região centro-oeste do ES. Ainda não existem serviços de saúde habilitados para realizar a coleta de vestígios da violência sexual, dificultando a estruturação da cadeia de custódia^(10)^.

Diante do exposto, frente aos danos da violência interpessoal à saúde é fortalecida a necessidade de realização de estudos sobre essa violência, que poderão auxiliar no processo de qualificação profissional e reestruturação de políticas públicas diante do cenário de enfrentamento às violências interpessoais. Portanto, o presente estudo teve por objetivo identificar a frequência de notificações de violência interpessoal no Espírito Santo, no período de 2011 a 2018, e os fatores associados a esse agravo.

## OBJETIVO

Identificar a frequência de notificações de violência interpessoal no Espírito Santo, no período de 2011 a 2018, e os fatores associados a esse agravo.

## MÉTODOS

### Aspectos éticos

Os dados analisados neste estudo são procedentes das Fichas de Notificação/Investigação de Violência Interpessoal e Autoprovocada, preenchidas por profissionais dos serviços de saúde privados e públicos, digitalizadas no Sistema de Informação de Agravos de Notificação (SINAN). O consentimento do paciente foi dispensado devido ao banco de dados ter sido concedido pelo setor de Vigilância Epidemiológica da Secretaria de Estado da Saúde do Espírito Santo. O estudo trabalha com dados secundários; contudo, por conter informações de identificação nominal no banco de dados cedido pela referida Vigilância Epidemiológica, o estudo passou pela aprovação do Comitê de Ética em Pesquisa da Universidade Federal do Espírito Santo no ano de 2018, seguindo a Resolução do Conselho Nacional de Saúde nº 466/12.

### Desenho e local do estudo

Foi realizado um estudo do tipo transversal. Aplicaram-se as diretrizes da rede EQUATOR por intermédio da ferramenta *Strengthening the Reporting of Observational Studies in Epidemiology* (STROBE)^(11)^. O cenário do estudo foi o estado do Espírito Santo, que abrange uma população de 3.514.952, possuindo um Índice de Desenvolvimento Humano (IDH) de 0,740 e uma densidade demográfica de 76,25 hab/km^2 (12)^.

### População; período; critérios de inclusão e exclusão

Neste estudo, foram incluídos todos os casos de violência interpessoal notificados no estado do Espírito Santo, no período de 2011 a 2018, a partir dos dados registrados no Sistema de Informação de Agravos Notificados (SINAN) cedidos pela Vigilância Epidemiológica do Espírito Santo. A escolha do período de estudo deve-se ao fato de que a violência foi inserida como um agravo de notificação compulsória a partir de 2011 ^(13)^. Não houve exclusão de casos; foram analisadas todas as fichas de notificação compulsória de violência interpessoal no período descrito no Espírito Santo.

Os dados registrados na ficha de notificação do SINAN e disponibilizados pela Secretaria de Estado da Saúde do Espírito Santo são procedentes dos atendimentos prestados pelos serviços de saúde públicos e privados. A compreensão das características analisadas na ficha de notificação compulsória de violência segue as diretrizes do Instrutivo de Notificação Interpessoal e Autoprovocada ^(13)^.

### Variáveis

A variável dependente deste estudo foi a violência interpessoal (analisada de forma dicotômica - sim/não), independentemente da sua natureza. Neste estudo, considera-se a violência interpessoal como a determinada no Instrutivo de Notificação Interpessoal e Autoprovocada, que se distingue em duas tipologias: violência doméstica/intrafamiliar, identificada como a que ocorre entre parceiros íntimos ou membros da família nos domicílios ou fora deles, e violência extrafamiliar/comunitária, que acontece no ambiente social abrangendo tanto conhecidos quanto desconhecidos ^(13)^. O conceito envolve as diferentes formas de violência interpessoal como: física; psicológica/moral; sexual; tortura; tráfico de seres humanos; trabalho infantil; intervenção legal; financeira/econômica; e negligência/abandono ^(13)^.

Como variáveis independentes, foram analisadas as características da vítima, do agressor e da agressão. Dentre as características da vítima, encontram-se o sexo (masculino; feminino), a faixa etária (em anos: 0 a 9; 10 a 19; 20 a 59; 60 ou mais), a raça/cor (branca; preta/parda), a presença de deficiências/transtornos (não; sim) e a zona de residência (urbana/periurbana; rural).

Em relação às características do agressor, foram avaliadas a faixa etária (em anos: 0 a 24; 25 ou mais), o sexo (masculino; feminino), o vínculo com a vítima (parceiro íntimo atual ou ex; familiar; conhecido; desconhecido) e a suspeita de uso de álcool (não; sim).

No que tange à agressão, foram analisados o número de envolvidos (um; dois ou mais), o local da ocorrência (residência; via pública; outros), a presença de histórico de violência de repetição (não; sim) e o encaminhamento da vítima para outros serviços (não; sim).

### Análise dos resultados e estatística

Antes das análises, os dados passaram por um processo de qualificação, conforme orientações do Instrutivo de Notificação de Violência Interpessoal e Autoprovocada ^(13)^. Os casos com dados em branco ou ignorados foram excluídos das análises, motivo pelo qual o número total de indivíduos variou de acordo com a característica estudada. Posteriormente, os dados foram trabalhados no pacote estatístico Stata, versão 14.0, e os resultados foram apresentados por meio de frequência absoluta e relativa, bem como intervalos de confiança de 95%. Para a análise bivariada, foram utilizados o Teste do Qui-Quadrado de Pearson e, no caso da análise multivariada, a Regressão de Poisson, com estimativa das razões de prevalência. Foi utilizado o modelo hierárquico, considerando as características da vítima como primeiro nível e as variáveis relacionadas ao agressor e ao evento como segundo nível. A entrada das variáveis no modelo respeitou o critério de p < 0,20 na análise bivariada, e sua manutenção se deu com p < 0,05. As variáveis “vínculo com a vítima” e “número de envolvidos” não foram incluídas nas análises bi e multivariada, pois o grupo de comparação são as notificações de violência autoprovocada.

## RESULTADOS

No período de 2011 a 2018, no Espírito Santo, foram notificados 27.277 casos de violência interpessoal, representando uma frequência de 75% (IC95%: 74,5-75,4) dentre todas as notificações realizadas no período (dados não apresentados em tabela).

Em relação à caracterização das vítimas, 75,0% eram mulheres (n=20.449), adultos (59,4%; n=16.191), de raça/cor preta/parda (71,0%; n=16.928), aqueles que não possuíam deficiência ou transtorno (90,7%; n=21.362) e que residiam em área urbana/periurbana (90,3%; n=24.129). Quanto ao agressor, nota-se que a maioria estava na faixa etária de 25 anos ou mais (68,9%; n=10.091), eram homens (82,9%; n=19.054), com vínculo de parceiro atual ou ex com a vítima (40,6%; n=9.811) e sem suspeita do uso de álcool durante o evento (54,5%; n=7.647). No que tange ao agravo, observa-se que a maioria foi cometida por um agressor (80,5%; n=19.628), nas residências (66,6%; n=15.853), sendo de repetição (54,5%; n=11.293), e, na maioria dos casos (84,9%; n=21.895), houve encaminhamento (Tabela 1).

**Tabela 1:** Características dos casos notificados de violência interpessoal. Espírito Santo, Brasil, 2011-2018

Variáveis	n	%	IC 95%^^*^^
Sexo			
Masculino	6828	25,0	24,5-25,6
Feminino	20449	75,0	74,5-75,5
Faixa etária			
0 a 9 anos	3060	11,2	10,9-11,6
10 a 19 anos	6348	23,3	22,8-23,8
20 a 59 anos	16191	59,4	58,8-59,9
60 anos e mais	1678	6,1	5,9-6,4
Raça/cor			
Branca	6911	29,0	28,4-29,6
Preta/parda	16928	71,0	70,4-71,6
Deficiências/transtornos			
Não	21362	90,7	90,3-91,1
Sim	2191	9,3	8,9-9,7
Zona de residência			
Urbana/periurbana	24129	90,3	89,9-90,6
Rural	2603	9,7	9,4-10,1
Faixa etária do agressor			
0 - 24 anos	4546	31,1	30,3-31,8
25 anos ou mais	10091	68,9	68,2-69,7
Sexo do agressor			
Masculino	19054	82,9	82,4-83,4
Feminino	3938	17,1	16,7-17,6
Vínculo com a vítima			
Parceiro íntimo atual ou ex	9811	40,6	39,9-41,2
Familiar	5132	21,2	20,7-21,7
Conhecido	5772	23,9	23,3-24,4
Desconhecido	3475	14,3	13,9-14,8
Suspeita de uso de álcool			
Não	9163	54,5	53,8-55,3
Sim	7647	45,5	44,7-46,3
Número de agressores			
Um	19628	80,5	80,0-81,0
Dois ou mais	4762	19,5	19,0-20,0
Local de ocorrência			
Residência	15853	66,6	66,0-67,2
Via pública	5121	21,5	21,0-22,1
Outros	2823	11,9	11,5-12,3
Violência de repetição			
Não	9445	45,5	44,9-46,2
Sim	11293	54,5	53,8-55,1
Encaminhamento			
Não	3900	15,1	14,7-15,6
Sim	21895	84,9	84,4-85,3

* *IC95%: Intervalo de Confiança de 95%; Fonte: Sistema de Informação de Agravos e Notificação (SINAN), de 2011 a 2018.*

Na análise bivariada, observa-se que o desfecho esteve associado a maioria das variáveis independentes em estudo, com exceção para o sexo da vítima, zona de residência e repetição do evento (Tabela 2).

**Tabela 2:** Análise bivariada da distribuição das características segundo às notificações de violência interpessoal. Espírito Santo, Brasil, 2011-2018

Variáveis	n	%	IC 95%^*^	Valor de *p*
Sexo				
Masculino	6828	75,2	74,3-76,1	0,493
Feminino	20449	74,9	74,4-75,4	
Faixa etária				
0 a 9 anos	3060	98,8	98,4-99,1	<0,001
10 a 19 anos	6348	72,1	71,1-73,0	
20 a 59 anos	16191	71,9	71,3-72,4	
60 anos e mais	1678	85,8	84,2-87,3	
Raça/cor				
Branca	6911	71,3	70,4-72,2	<0,001
Preta/parda	16928	78,0	77,4-78,6	
Deficiências/transtornos				
Não	21362	82,6	82,1-83,0	<0,001
Sim	2191	48,2	46,7-49,6	
Zona de residência				
Urbana/periurbana	24129	74,8	74,3-75,3	0,217
Rural	2603	75,8	74,3-77,2	
Faixa etária do agressor				
0-24 anos	4546	55,0	53,9-56,1	<0,001
25 anos e mais	10091	70,5	69,8-71,3	
Sexo do agressor				
Masculino	19054	88,9	88,5-89,3	<0,001
Feminino	3938	38,0	37,1-39,0	
Suspeita de uso de álcool				
Não	9163	65,5	64,7-66,3	<0,001
Sim	7647	86,2	85,5-86,9	
Local de ocorrência				
Residência	15853	68,7	68,1-69,3	<0,001
Via pública	5121	92,5	91,8-93,2	
Outros	2823	85,3	84,1-86,5	
Violência de repetição				
Não	9445	74,7	74,0-75,5	0,160
Sim	11293	75,5	74,8-76,1	
Encaminhamento				
Não	3900	69,6	68,4-70,8	<0,001
Sim	21895	76,1	75,6-76,6	

* *IC95%: Intervalo de Confiança de 95%; Fonte: Sistema de Informação de Agravos e Notificação (SINAN), de 2011 a 2018.*

Observa-se na análise multivariada, após o controle dos fatores de confusão, que crianças e idosos tiveram 29% e 25%, respectivamente, mais prevalência de notificação da violência interpessoal quando comparados aos adolescentes. Outro achado foi uma maior frequência de notificação em indivíduos de raça/cor preta/parda (RP: 1,07; IC95%: 1,06-1,09), em comparação aos que se declararam brancos. Ainda, nota-se que as notificações de violência interpessoal foram 69% maiores nas pessoas sem deficiência. Quanto ao perpetrador, verifica-se uma maior prevalência de pessoas com 25 anos ou mais (RP: 1,15; IC95%: 1,13-1,18), sendo duas vezes mais homens (RP: 2,09; IC95%: 2,01-2,17) e com suspeita de uso de álcool no momento da agressão (RP: 1,12; IC95%: 1,10-1,14). No que tange ao agravo, a via pública foi o local de maior ocorrência das violências notificadas (RP: 1,32; IC95%: 1,29-1,35), e apresentou caráter de repetição (RP: 1,09; IC95%: 1,07-1,11) (Tabela 3).

**Tabela 3:** Modelo multivariado com a razão de prevalência bruta e ajustada das variáveis associadas a notificação de violência interpessoal. Espírito Santo, Brasil, 2011-2018

Variáveis	Análise bruta			Análise ajustada		
	RP^*^	IC 95%^†^	Valor de *p*	RP^*^	IC 95%^†^	Valor de *p*
Faixa etária						
0 a 9 anos	1,37	1,35-1,39	<0,001	1,29	1,27-1,30	<0,001
10 a 19 anos	1,0			1,0		
20 a 59 anos	0,99	0,98-1,01		1,04	1,02-1,05	
60 anos e mais	1,19	1,17-1,22		1,25	1,22-1,28	
Raça/cor						
Branca	1,0		<0,001	1,0		<0,001
Preta/parda	1,10	1,08-1,11		1,07	1,06-1,09	
Deficiências/transtornos						
Não	1,72	1,66-1,77	<0,001	1,69	1,64-1,75	<0,001
Sim	1,0			1,0		
Faixa etária do agressor						
0-24 anos	1,0		<0,001	1,0		<0,001
25 anos e mais	1,28	1,25-1,31		1,15	1,13-1,18	
Sexo do agressor						
Masculino	2,34	2,28-2,40	<0,001	2,09	2,01-2,17	<0,001
Feminino	1,0			1,0		
Suspeita de uso de álcool						
Não	1,0		<0,001	1,0		<0,001
Sim	1,32	1,30-1,34		1,12	1,10-1,14	
Local de ocorrência						
Residência	1,0		<0,001	1,0		<0,001
Via pública	1,35	1,33-1,36		1,32	1,29-1,35	
Outros	1,24	1,22-1,26		1,22	1,19-1,26	
Violência de repetição						
Não	1,0		0,160	1,0		<0,001
Sim	1,01	0,99-1,02		1,09	1,07-1,11	

* RP: Razão de Prevalência;

† IC95%: Intervalo de Confiança de 95%; Fonte: Sistema de Informação de Agravos e Notificação (SINAN), de 2011 a 2018.

## DISCUSSÃO

O presente estudo identificou uma elevada frequência de notificação de violência interpessoal no Espírito Santo no período de 2011 a 2018, dado que corrobora com pesquisa realizada no Paraná, em que a prevalência desse agravo também representou a maioria dos casos de notificação (90%) comparado à violência autoprovocada (10%) ^(14)^. Em números absolutos, nesse estudo realizado no Paraná, a população mais afetada por violência interpessoal foi a de crianças; já no presente estudo, a maior ocorrência dos casos de notificação de violência interpessoal se concentrou entre mulheres na fase adulta. Contudo, sob a ótica de associação estatística, as crianças de 0-9 anos de idade apresentaram uma prevalência 29% maior quando comparadas aos adolescentes de 10-19 anos de idade, enquanto os adultos de 20-59 anos de idade apresentaram uma prevalência 4% maior quando comparados aos adolescentes^(14)^.

Nota-se ainda uma maior prevalência de notificações de violência interpessoal contra crianças. É importante ponderar que essa população se encontra em maior grau de dependência com sua família, que tanto pode oferecer apoio e segurança, quanto se ausentar de sua responsabilidade, privando a criança de seus direitos e, assim, prejudicando o seu desenvolvimento nas esferas física, social, comportamental, emocional e cognitiva ^(15)^. Ainda sobre o estudo realizado no Paraná, foi observado que o tipo mais frequente de violência interpessoal notificado entre crianças foi o de negligência/abandono ^(14)^. Em um estudo realizado com análise de notificações de violência no Espírito Santo, identificou-se que os pais/padrastos/mães/madrastas são os agressores mais prevalentes quando se trata de negligência/abandono ^(16)^, e os principais motivos para a ocorrência das violências contra pessoas nessa faixa etária são: baixa renda familiar, baixo nível de instrução, pobreza e falta de planejamento familiar^(17)^.

Verifica-se na presente pesquisa a maior prevalência de notificação de violência interpessoal entre indivíduos de raça/cor preta/parda, o que está em consonância com um estudo brasileiro ^(18)^. A população negra é a maior vítima das iniquidades socioeconômicas, cujas justificativas se referem às desigualdades sociais, preconceito e discriminação presentes na sociedade. Esse contexto se torna um terreno fértil para a ocorrência das violências ^(19)^. Uma importante visão sobre a relação entre as violências e a população negra é a lente teórica da interseccionalidade, uma forma de observar as consequências estruturais e dinâmicas da relação entre dois ou mais eixos da subordinação, tais como a interação do racismo com outros eixos. Com esse olhar interseccional, fica mais nítido o cenário de vulnerabilidades sociais que expõem a população negra a uma frequência maior de situações de violência^(20)^.

Interessante destacar que as pessoas sem deficiência foram as mais prevalentes entre os casos notificados neste estudo. Dados do inquérito VIVA de 2017 destacaram uma frequência de 95,7% de agressões perpetradas contra pessoas sem deficiência ^(21)^. Entretanto, vale mencionar os desafios envolvidos na identificação e reconhecimento de atitudes violentas cometidas contra pessoas com deficiência. Entre os anos de 2009 e 2013, o Brasil apresentou um aumento de 7,1% nos casos notificados de violência interpessoal contra essa população ^(22)^. Dificuldades relacionadas à conceituação do termo deficiência, assim como a falta de visibilidade da vítima devido a suas limitações e as relações de poder estabelecidas por familiares e cuidadores, favorecem o distanciamento de medidas de proteção e denúncia ^(23)^.

Foi identificado neste estudo que há uma maior ocorrência de casos de violência envolvendo agressores adultos e do sexo masculino. Esse achado também foi observado em um estudo que analisou as notificações de violência interpessoal realizadas em todo o Brasil entre o período de 2015 a 2019, no qual encontrou uma prevalência de 54% de agressores do sexo masculino e 45% de agressores adultos^(17)^. Nesse cenário, é importante considerar que homens autores de violência sustentam um discurso de domínio sobre a mulher, a provisão do lar, a chefia familiar e a sexualidade exacerbada. Tais atributos sociais da masculinidade alimentam a prática da violência doméstica. Discursos que os homens autores de violência elencaram como características intrínsecas e indispensáveis sobre como se constituir como homem, de modo que qualquer conduta de insubordinação da mulher ou menina que ameaçasse a manutenção desse modelo de masculinidade, é considerada um gatilho para a prática da violência ^(24)^.

O uso de álcool pelo agressor no momento da agressão representou um aumento de 12% na prevalência da violência interpessoal quando comparados aos que não consumiram bebida alcoólica, achado também encontrado em outro estudo ^(25)^. Para a Unidade de Pesquisa em Álcool e Drogas da Universidade Federal de São Paulo, o consumo de álcool por parte do agressor, mesmo de forma não abusiva, tem papel significativo na perpetração de violência ^(26)^. Além disso, o álcool desempenha um importante papel na desinibição fisiológica, tendo também um efeito sobre a expectativa das outras pessoas em aceitar tais comportamentos, frequentemente resultando em comportamento violento ^(27,28)^. O uso de álcool está associado, ainda, a uma maior gravidade das consequências da violência por parceiro íntimo, achado que está em concordância com estudos tanto nacionais quanto internacionais ^(27-29)^.

Quanto ao local de ocorrência dos casos notificados, a via pública apresentou a maior frequência geral dos casos notificados de violência interpessoal, corroborando com os achados de um estudo que analisou 4.406 casos de violência que ocorreram em capitais brasileiras ^(30)^. Contudo, esse mesmo estudo aponta que há diferenciação no perfil do local de ocorrência quando há uma análise estratificada, principalmente quanto ao gênero e idade.

Os espaços públicos (ruas, bares e outros locais) representam o principal cenário para a ocorrência de eventos violentos, principalmente em relação a agressores e vítimas do sexo masculino. O domicílio é o espaço onde ocorre a maioria das violências contra mulheres, crianças, adolescentes e idosos, pois é onde passam a maior parte do seu tempo e onde vivem a maioria dos agressores. Qualquer membro da família pode se tornar, em determinada circunstância, vítima e agressor ^(30)^.

Após análise de correspondência simples, a via pública permaneceu associada ao evento e não à residência ^(31)^. De todo modo, é importante destacar a maior vulnerabilidade das vítimas em ambientes domésticos, podendo estas permanecerem silenciadas por seus agressores e só vistas em casos de exposição em ambientes externos, visto que a notificação da violência se dá quando o serviço tem contato com a vítima, sendo uma limitação, visto que nem todos os casos possuem contato com um serviço notificador (serviço de saúde público ou privado, escolas) ^(2,23)^.

Vale ponderar o caráter de repetição dos casos notificados de violência interpessoal apresentados nos achados do presente estudo, dado que se assemelham a estudos realizados por Leite ^(32)^. A repetição muitas vezes está associada ao convívio próximo com o agressor, por vezes familiar e na residência, o que pode contribuir para a demora na procura por serviços de saúde ^(33)^. Além disso, quando se trata de violência psicológica e negligência, torna-se difícil seu registro devido à subjetividade, contribuindo para que a repetição ocorra ^(34)^. Além disso, a vítima tem de lidar com o constrangimento, o medo da humilhação e da incompreensão que fazem com que muitas vezes a culpa recaia sobre ela, o que contribui para que a denúncia não ocorra, ampliando as chances de recorrência da violência. Consequentemente, ao continuar nesse contexto violento, também se encontram em maior risco de sofrer desordens alimentares, alcoolismo e abuso de outras drogas, estresse pós-traumático, depressão, ansiedade, fobias, pânico e baixa autoestima ^(33,35)^.

Por sua vez, como um problema complexo que demanda a atuação de diversos setores para seu enfrentamento, a saúde precisa atuar em rede, de forma articulada, sendo imprescindível um trabalho em equipe multidisciplinar. No que se refere à enfermagem, esta possui uma ferramenta importantíssima para detectar os casos de violência: as consultas, em que se realiza a anamnese e o exame físico. É importante deixar claro que, durante a anamnese, alguns aspectos do discurso e do comportamento são indicadores de suspeita de violência para os profissionais da saúde ^(36)^.

Assim, as atribuições primordiais do(a) enfermeiro(a), principalmente na atenção primária, são as seguintes: planejamento de ações em promoção da saúde, prevenção de ações violentas e agravos à saúde e, finalmente, a interação com outras equipes, a fim de que a vítima receba atenção integral^(37)^. Ademais, é atribuição dos profissionais de saúde identificar e notificar os casos suspeitos ou confirmados de violência. Sendo relevante a capacitação permanente sobre o tema e a viabilização de ferramentas que facilitem e qualifiquem o processo de notificação, como um sistema on-line, pode ser relevante para aprimoramento da notificação enquanto importante dimensão da linha de cuidado ^(5)^.

É nesse processo educativo e de conscientização que os enfermeiros, enquanto membros da equipe de saúde e sujeitos multiplicadores de conhecimentos, são de suma importância, pois, na promoção de educação em saúde com a população, proporcionarão o conhecimento necessário para despertar na população o interesse e desmistificar o medo relacionado às denúncias ^(38)^.

Ainda, é essencial destacar a potência da abordagem multiprofissional aos casos de pessoas em situação de violência interpessoal. Nessa abordagem, um fator importante é que os profissionais estejam capacitados para utilizar as informações adquiridas e observadas durante a identificação e pós-identificação de violências, para que dessa forma não cometam outras violências diante da vulnerabilidade da vítima, tanto nas demandas de saúde das pessoas em situação de violência quanto no atendimento intersetorial que objetivam proporcionar os cuidados e proteção necessários, variando conforme as complexidades e particularidades de cada caso ^(39)^. O despreparo da equipe multiprofissional na identificação e no manejo dos casos de violência pode auxiliar na perpetuação da situação de violência, favorecendo a manutenção ou a piora do cenário, expondo a pessoa a um contexto que favorece a repetição dos atos violentos ^(40)^.

Dado o cenário da relevância da notificação das violências, é válido reforçar que a notificação é uma das frentes da Linha de Cuidado para atenção integral à saúde das pessoas, sejam crianças, adultos, idosos, homens, mulheres ou demais componentes de uma família, em situação de violência. Essa linha, além de monitorar as atividades e prevenir as violências, também atua em acolhimento, atendimento, cuidados profiláticos, tratamento, seguimento na rede de cuidado, proteção social, e promoção da saúde e da cultura da paz ^(41,42)^. Acompanhando nesses trâmites as legislações que preveem assistências e direitos para os grupos específicos, como por exemplo: Estatuto da Criança e do Adolescente (ECA), Estatuto do Idoso, Estatuto da Pessoa com Deficiência, normas técnicas do Ministério da Saúde, entre outros ^(41)^. Dessa forma, reitera-se a magnitude da notificação como uma ferramenta de cuidado e garantia de direitos.

### Limitações do estudo

No que tange às limitações deste estudo, uma delas diz respeito à utilização de dados obtidos no SINAN, sujeitos à incompletude das informações ou subnotificação. Visto que as fichas de notificação de violência são preenchidas nos serviços de saúde, o profissional precisa ser capacitado para reconhecer e notificar o caso suspeito ou confirmado. Nesse sentido, é reconhecida a subnotificação dos casos de violência, especialmente das lesões autoprovocadas, não somente pelo estigma desse agravo, mas também pelas fragilidades do sistema de vigilância em saúde ^(43)^. Outra limitação do estudo é o seu delineamento, que não permite estabelecer relações de causalidade considerando que os dados são coletados em um único momento.

### Contribuições para a área

Os achados do presente estudo poderão auxiliar na elaboração ou reestruturação de políticas públicas de enfrentamento às diferentes formas de violência interpessoal. No mesmo sentido, os resultados podem ampliar a visão dos profissionais de saúde quanto ao cuidado com a população mais vulnerável à violência interpessoal, estimulando intervenções de prevenção e promoção contra a ocorrência desse fenômeno, bem como reforçar a importância da qualificação dos profissionais, refletindo assim no melhor preenchimento dos dados, lançamento no sistema e na redução da incompletude da ficha de notificação.

## CONCLUSÕES

No Espírito Santo, no período de 2011 a 2018, os resultados demonstram um número elevado de casos notificados de violência interpessoal. Todavia, deve-se considerar a presença de subnotificação, o que acarretaria um número maior de vítimas, fato preocupante que leva a constatar a importância de profissionais qualificados e aptos ao rastreio de sinais e sintomas sugestivos de violência durante os atendimentos nos serviços de saúde, e, consequentemente, à notificação e manejo dos casos.

Outro ponto a destacar é a maior vulnerabilidade de crianças e idosos à violência interpessoal, assim como os homens constituírem os principais perpetradores. Tais achados revelam o quanto a violência está atrelada às relações de desigualdade de poder, sendo de grande importância que a temática de igualdade de gênero seja abordada nos diferentes cenários de formação do indivíduo, como na família, escola e nos serviços de saúde.

Por fim, e não menos importante, foi verificar que a vitimização apresentou um caráter recorrente. Achado extremamente preocupante, pois aponta o quanto o rastreio precoce da violência ainda é falho e o quanto a vítima permanece nesse ciclo por repetidas vezes. Os serviços de saúde e demais espaços de assistência devem buscar um rastreio precoce da violência e, assim, oportunizar a inserção da vítima na rede de cuidado e proteção.

Assim, com base nos dados encontrados no presente estudo, percebe-se a necessidade de fortalecimento das redes de ação intersetorial para proteção e atendimento às vítimas, considerando o caráter multidimensional da violência, além do fortalecimento das políticas públicas, visando garantir a sua efetividade e a proteção dos segmentos mais vulneráveis da sociedade.
